# Notes and Notices

**Published:** 1899-10

**Authors:** 


### NOTES AND NOTICES.

Dr. E. Mather formerly of Detroit, Michigan, has removed to Birmingham, Oakland County, Michigan.

See a lot of Portuguese men in another column treading grapes with their feet for making wine. Read about it, also about Speer's improved method of mashing grapes and making wine.

Bacteriology as a Fad.—The following remarkable statement appears in *The Home Magazine:* "Dr. Heneage Gibbes, after occupying the chair of bacteriology for ten years or more at Ann Arbor has accepted the position of health officer for the city of Detroit. Dr. Gibbes to-day denies bacteria as a causative factor in disease. It has to be borne in mind, too, that in publishing this statement he does not give it as his opinion merely, but as a fact based upon actual experiments; said experiments, consisting of inoculation with bacteria, having been performed upon himself time and again without the slighest effect. He not only says that the idea of dodging a bacillus here for one thing and another somewhere else for another thing, is absurd and 'simply a fad,' but he absolutely denies the fact that—hitherto urged as a proof of their etiological nature—that these 'pathogenetic' micro-organisms are always present in disease. He says: 'I have conducted hundreds of autopsies on consumptives without finding a trace of the bacillus tuberculosis. I taught along the lines of Koch, but presented results for what they were worth only. There is no such thing as *German* science, science is universal. My personal investigations have convinced me that the whole germ theory of disease is a fad. In Germany, Dr. Koch's theories are regarded as *theories* only; but in this country they are held too frequently to be *facts.*' "—*Medical Times.*

Noah after the flood planted the first fruit, the grape, the most healthy of all the products of the earth.

### CASE OF VIRULENT CHRONIC CATARRH CURED.

DR. S——, NEW YORK.

Margie Bowers, age 24; January 11th, 1897; chronic rhinitis, from scrofulous diathesis; mucous membrane much thickened and dark red; a number of polypoid growths; considerable ulceration and loss of structure; thick, tough, greenish, and fetid secretion; large accumulations of dried mucus; sense of smell entirely lost, and hearing nearly so; constant frontal headache; congestion of the eyes; in fine, profound general anæmia.

All the known treatments were pursued, with little or no relief. But on January 16th, at the suggestion of a professional brother, I determined to test the blood cure, and commenced by giving bovinine every two hours. The nasal passages were thoroughly cleansed with Thiersch solution; the polypoid growths were snared off, and the points of ulceration were touched up with 25 per cent. pyrozone. BOVININE in salt water. was sprayed into the passages every two hours; preceded every time by washing out with Thiersch solution.

The effect of this course of treatment, continued, can only be described as magical. Within the first forty-eight hours, the before constant headache ceased; the discharge was diminished, and its fetid odor had disappeared. January 28th, the sense of smell had returned, and the hearing was improved. February 20th, the points of ulceration was entirely healed and all symtoms were practically normal.—*Handbook of Hæmatherapy.*

FOR THE WEAK AND AGED.—The best thing for weakly persons and invalids is Speer's Port Grape Wine. His Burgundy and Claret Wines are used at dinner by the best society people in New York and Washington.

### OLD CHRONIC NASO-PHARYNGEAL CATARRH.

DR. J——, NEW YORK.

John Bostwick; age 22; June 10th, 1897; old chronic case, anterior nares showing three distinct points of ulceration; the posterior nares tremendously hypertrophied, occluding the passages almost entirely; tonsils hypertrophied; fauces covered with points of ulceration; large accumulations of fetid mucus and breath so foul that he never went anywhere among others; had been treated by several of the best specialists, with but slight and temporary relief. In my judgment an operation was indispensable, and I so advised; but he refused to submit to any operation whatever. My former success with bovine blood encouraged me to try it; spraying bovinine into the nasal passages and throat, following it with a spraying of peroxide, and then washing out the product with Thiersch. The patient was directed to apply iodoform-bovinine with a medicine-dropper, and to swab the throat with the same, three times a day, and return every morning for the bovinine-peroxide treatment. July 17th, all points of ulceration were healed, and the patient was relieved of the distressing accumulation of mucus. The sense of smell was regained, and the hearing almost entirely restored.—*Handbook of Hæmatherapy.*

The Frequency of Left-handedness.—Among other peculiar observations made by Dr. Hrdlicka, in recent studies upon anthropology, were those made to ascertain the number of left-handed individuals among the children. There is no particular significance in the simple fact that a person is left-handed, or, at least, we know as yet positively of no such significance, and the investigations as to this point have up to now been largely only statistical. Among the 1,000 inmates of the asylum where the investigation was made there were six left-handed boys and four left-handed girls. In some of these subjects the left-handedness probably was more apparent than real, as in two of the boys and three of the girls, notwithstanding the left-handedness, the right arm was found to be the strongest.—*Archives of Pediatrics,* August, 1899.

Speer, the oldest wine grower in the United States, has vineyards of the Portugal Grape, from which his wines are made and fully matured by great age, and are valuable.

---

### FUN FOR DOCTORS.

### The Doctor as a Biographer.

Visitor.—"Why did you say that gentleman who went out was your biographer? I am sure it was Dr. Jones.

Invalid.—"Same thing! He is at work on my life."—*Medical Record.*

### Tumors.

Doctor (Ear at patient's chest)—"This swelling here must be reduced at once."

Patient.—"Go slow, Doc, that swelling happens to be my pocket-book!"—*Truth.*

### Worse Than the Disease.

"Yes," said the physician, "I will not only cure you, but I will also put your portrait in the papers."

"Then let me die!" exclaimed the unhappy man in a voice of anguish.

### Took Him at His Word.

"What you n ed," observed the doctor, "is change."

"Exactly," replied the patient. "I've left all mine at home; you must allow me to be in your debt for this visit."

But this wasn't the doctor's meaning at all.

### No Laughing Matter.

"The ills of some of your patients are wholly imaginary, are they not, doctor?"

"They are."

"I suppose you have many a good laugh over them."

"Not at all, my dear sir. I do not laugh at that which brings me my bread and butter."—*New York Press.*

